# P-1964. Tendon Rupture and Musculoskeletal Events Associated with Fluoroquinolones: A Longitudinal FAERS Review

**DOI:** 10.1093/ofid/ofaf695.2131

**Published:** 2026-01-11

**Authors:** Dona Ann Varkey, Jose J Kochuparambil

**Affiliations:** Mary Queens Mission Hospital, Anakkallu, Kerala, India; MQMH, Pala, Kerala, India

## Abstract

**Background:**

Fluoroquinolones are broad-spectrum antibiotics frequently used in outpatient and hospital settings. However, growing evidence has linked them to musculoskeletal adverse events (AEs), particularly tendon rupture. These concerns have prompted regulatory warnings, yet real-world reporting patterns and signal strength remain under-characterized. This study aims to evaluate the disproportionality and trends of tendon and musculoskeletal events associated with fluoroquinolone use in the U.S. FDA Adverse Event Reporting System (FAERS).

**Figure:**
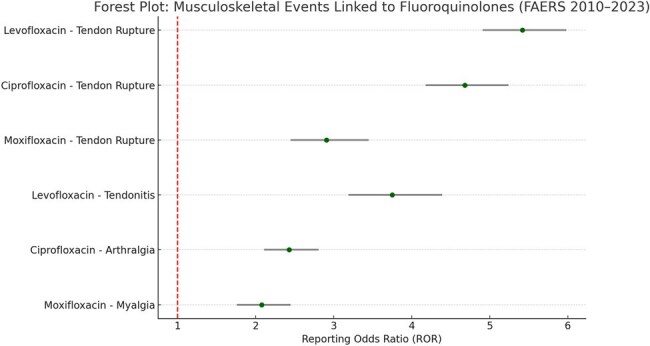
Forest Plot: Musculoskeletal Events Linked to Fluoroquinolones (FAERS 2010–2023) This forest plot displays Reporting Odds Ratios (RORs) with 95% confidence intervals for fluoroquinolone-associated musculoskeletal adverse events. Levofloxacin and ciprofloxacin showed strong signals for tendon rupture (ROR: 5.42 and 4.68, respectively), while moxifloxacin demonstrated a lower but significant signal (ROR: 2.91). Additional signals for tendonitis, arthralgia, and myalgia support ongoing concerns about fluoroquinolone-induced connective tissue toxicity.

**Methods:**

A retrospective longitudinal analysis was conducted using FAERS data from January 2010 to December 2023. Reports listing ciprofloxacin, levofloxacin, or moxifloxacin as the primary suspect drug were included. Musculoskeletal-related MedDRA Preferred Terms were extracted, including tendon rupture, tendonitis, arthralgia, and myalgia. Disproportionality was assessed using Reporting Odds Ratios (RORs) with 95% confidence intervals (CI). Year-wise trends in event frequency were analyzed to observe the impact of safety warnings.

**Results:**

A total of 9,837 musculoskeletal AE reports were linked to fluoroquinolone use. Tendon rupture was the most reported serious AE, with levofloxacin showing the strongest signal (ROR: 5.42; 95% CI: 4.91–5.98), followed by ciprofloxacin (ROR: 4.68) and moxifloxacin (ROR: 2.91). Tendonitis and arthralgia were also frequently reported. A marked decline in reporting frequency was observed post-2018, coinciding with updated FDA black box warnings. Elderly patients and those on corticosteroids had disproportionately higher reports of rupture events.

**Conclusion:**

This FAERS-based longitudinal review confirms a strong and consistent safety signal linking fluoroquinolones to tendon rupture and related musculoskeletal events. Findings reinforce the need for prescriber caution, especially in high-risk populations, and underscore the value of pharmacovigilance systems in monitoring evolving safety concerns.

**Disclosures:**

All Authors: No reported disclosures

